# Three-dimensional spatiotemporal tracking of fluorine-18 radiolabeled yeast cells via positron emission particle tracking

**DOI:** 10.1371/journal.pone.0180503

**Published:** 2017-07-06

**Authors:** Seth T. Langford, Cody S. Wiggins, Roque Santos, Melinda Hauser, Jeffrey M. Becker, Arthur E. Ruggles

**Affiliations:** 1 Department of Nuclear Engineering, University of Tennessee-Knoxville, Knoxville, Tennessee, United States of America; 2 Department of Physics and Astronomy, University of Tennessee-Knoxville, Knoxville, Tennessee, United States of America; 3 Departamento de Ciencias Nucleares, Escuela Politécnica Nacional, Quito, Ecuador; 4 Department of Microbiology, University of Tennessee-Knoxville, Knoxville, Tennessee, United States of America; University of Chicago, UNITED STATES

## Abstract

A method for Positron Emission Particle Tracking (PEPT) based on optical feature point identification techniques is demonstrated for use in low activity tracking experiments. A population of yeast cells of approximately 125,000 members is activated to roughly 55 Bq/cell by ^18^F uptake. An *in vitro* particle tracking experiment is performed with nearly 20 of these cells after decay to 32 Bq/cell. These cells are successfully identified and tracked simultaneously in this experiment. This work extends the applicability of PEPT as a cell tracking method by allowing a number of cells to be tracked together, and demonstrating tracking for very low activity tracers.

## Introduction

Positron Emission Particle Tracking (PEPT) was first proposed in a US Patent by Shaw in 1978 [1]. Shaw envisioned tracking “signaling particles” in hemodynamic flow and also laid out the strategy for locating particles activated with a positron emitter based on the collection of coincidence lines (CLs) formed by the coincident gamma rays created when the positron annihilates with an electron. The detection technology and isotope availability improved, and in 1993, Parker *et al*. [2] introduced PEPT for engineering flow assessment. Since that time PEPT has been used to evaluate flow in milling equipment [3], hydrocyclones [4], and dishwashers [5], among other engineering apparatuses but has not been extensively used for biomedical purposes. The majority of PEPT evaluations have used the Birmingham method [2] and tracked a single particle. This method was also extended to track up to three particles of very different activities [6]. Bickell *et al*. [7] introduced a separate method for PEPT processing that allowed for tracking multiple particles of known initial positions. Wiggins *et al*. [8] advanced this method to track an arbitrary number of particles of unknown initial positions. This has since been used to examine flows where particles routinely pass into and out of the field of view of the detection system [9, 10]. Another adaptation of the method of Bickell *et al*. is introduced in [11] and is described and used herein.

Past studies have been conducted in which positron emission tomography (PET) has been used for the imaging of large groups of cells [12–16]. However, conventional PET is not suited for the tracking of individual cells, as is PEPT. Lee *et al*. [17] explored tracking of a single cell using a PET scanner and a specialized particle-tracking algorithm that fits CL data to cubic splines. They demonstrated via a Monte Carlo simulation of a Siemens Inveon preclinical PET scanner [18] that single cell tracking should be possible when cell activity exceeds 10 Bq. The model was further validated with data using a 1000 Bq point source in a Siemens Inveon PET scanner. They also noted that the isotope ^176^Lu is present in the lutetium oxyorthosilicate (LSO) crystals of the Inveon PET scanner and causes a background count rate not represented in their Monte Carlo model. They concluded that tracking of low activity sources using the Inveon scanner, with intrinsic ^176^Lu decays causing random coincidence count rate near that associated with a 100 Bq positron emitting source, was not feasible [17] and have since showed the ability of their method to track low activity sources in a scanner without LSO [19].

Goertzen *et al*. [20] showed that using an energy window lower level discriminator of at least 400 keV in a microPET R4 with LSO crystals nearly removes the random coincidence count rate attributable to ^176^Lu decay. However, this restriction of the energy window causes some reduction in the scanner sensitivity. Data presented herein use a restricted energy window of 425–625 keV.

To date, the authors have observed no experimental tracking of individual cells via positron imaging in the literature. A yeast cell preparation and activation protocol is presented that achieves 6.88 MBq activity in a population of roughly 125,000 cells, corresponding to near 55 Bq /cell. Serial dilution isolated nearly 20 cells for *in vitro* imaging in a 500 mL bottle of water. We report our imaging protocol and PEPT method, along with yeast cell tracking results.

## Materials and methods

### PEPT method

In PEPT, particle positions are calculated based on an examination of CLs connecting coincident detection sites, sorted according to time. PEPT differs from PET reconstruction in that it assumes that all radiation is emitted from point-like sources. As such, it allows for the detection and location of small sources using far fewer coincidence events than conventional PET. Bickell *et al*. [7] developed a “line density” method in which the number of CL crossings is tallied across a 3D grid superimposed over the field of view (FOV) of the scanner, a process similar to an unfiltered back-projection [21]. A particle’s position is determined based on a 3D Gaussian fit to this line density grid about the element having the most CL crossings. This method was also used for tracking of multiple particles of known initial positions. Wiggins *et al*. [8] used G-means clustering [22] to extend this line density method to be able to track multiple particles without *a priori* knowledge of particle number or position.

Another adaptation of the line density method was developed by Wiggins *et al*. [11] and is used herein. A brief description follows. When considering the aforementioned grid of CL crossings, one can view each element of the grid as a 3D counterpart to the pixel called a “voxel”, with the number of line crossings being analogous to a greyscale value. As such, traditional optical particle tracking methods may be adapted for use in PEPT. The method used in this work is based on the feature point identification techniques of Crocker and Grier [23] and Sbalzarini and Koumoutsakos [24], and thus is referred to as the feature point identification (FPI) method.

First, CLs are grouped into time steps and CL crossings are tallied across a grid as described above. Given this, let *N*^*t*^*(x*,*y*,*z)* be the number of line crossings at position *(x*,*y*,*z)* during time frame *t*. The grid is first smoothed via convolution with a box-car kernel of width 2*f*+1:

$$N^{t}\prime \;(x,y,z)=\frac{1}{{\left(2f+1\right)}^{3}}\sum_{i=x-f}^{x+f}\sum_{j=y-f}^{y+f}\sum_{k=z-f}^{z+f}N^{t}(i,j,k)$$ (1)

where *f* is taken to be a smoothing size. In this study, *f* is set to 1 for all instances.

Next, particle positions are estimated as local maxima in the grid *N*^*t*^*’*. Local maxima are taken to be grid elements having CL crossing values in the upper *r*^th^-percentile of their given frame and having values greater than any of their neighbors within a cube of side width *2w+1*. This parameter *w* serves as an apparent particle radius where each virtual particle image can be viewed as region of high CL density. However, *w* also serves as a particle resolving limit and can be set according to expected particle density.

Position refinements are then made according to centroid calculations over a cube of side width *2w+1* about each estimate. This cubic region serves as a virtual particle image. Given an estimate position of ***x*** *= (x*, *y*, *z)*^*T*^, a final position, ***x’*** *= (x’*, *y’*, *z’)*^*T*^ is calculated by

$$\left(\begin{array}{c} x\prime \\ y\prime \\ z\prime \end{array}\right)=\frac{1}{K}\sum_{i=x-w}^{x+w}\sum_{j=y-w}^{y+w}\sum_{k=z-w}^{z+w}\left(\begin{array}{c} i\\ j\\ k \end{array}\right)N^{t}\prime \;(i,j,k)$$ (2)

with normalization:

$$K=\sum_{i=x-w}^{x+w}\sum_{j=y-w}^{y+w}\sum_{k=z-w}^{z+w}N^{t}\prime \;(i,j,k).$$ (3)

**Fig 1:**
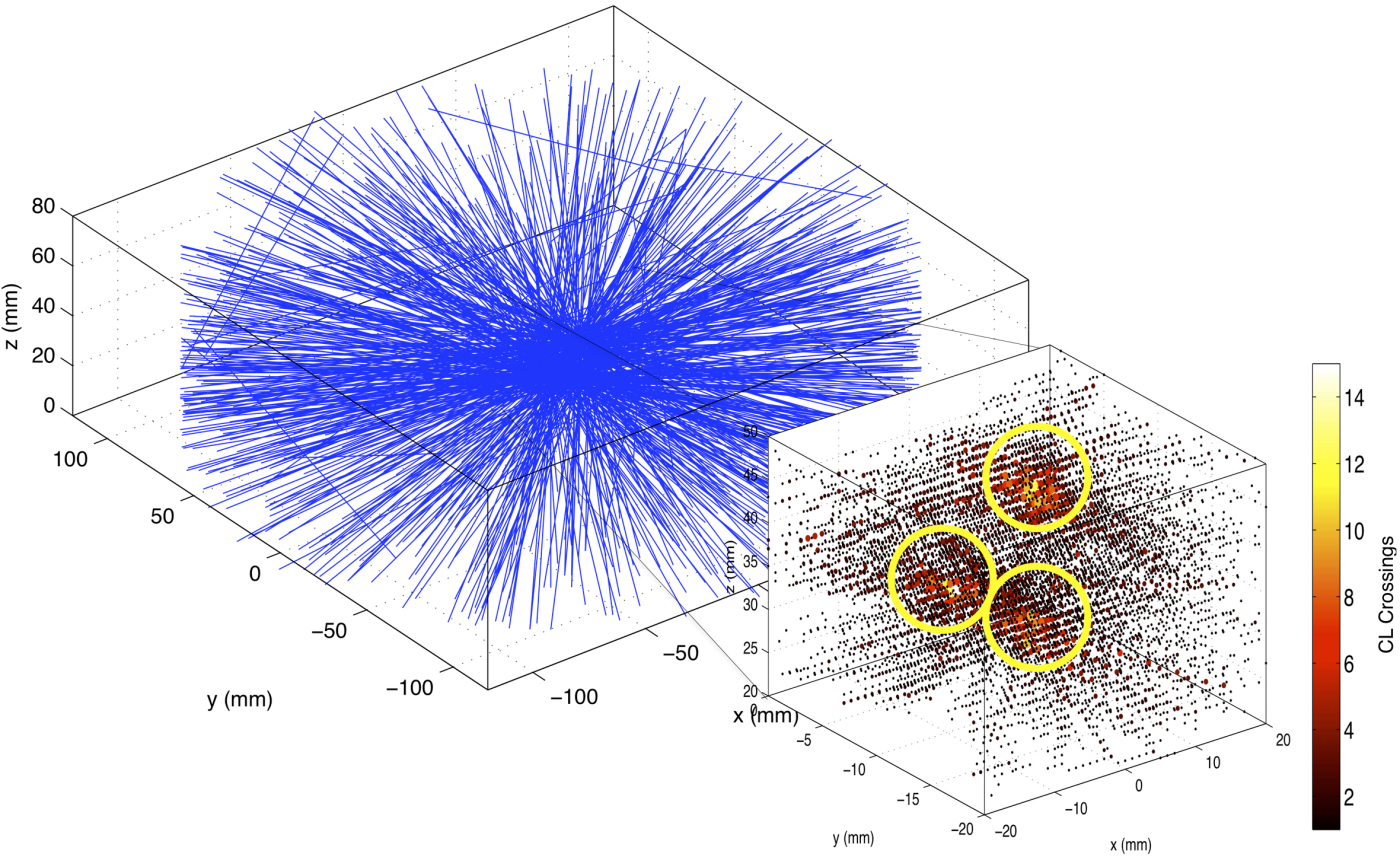
Coincidence lines and line density grid from sample measurement. Coincidence lines are generated from 10 msec scan of three 1.5 MBq particles. Inset is the line density tallying of this data over grids of size 1 mm × 1 mm × 1 mm. Local maxima are circled.

A pictorial representation of this process is seen in Fig 1. Here a collection of CLs from a 10 msec frame of three particles of activity 1.5 MBq each near the center of FOV of a MicroPET P4 scanner is shown. Inset is a line density tallying of this dataset with CLs counted over grids of size 1 mm × 1 mm × 1 mm. The grid shown is not smoothed. Identified local maxima are circled in yellow. Final positions are determined via centroid calculations about these maxima. Individual particle positions are then linked into trajectories via a two-frame, nearest-neighbors method, as described in [8].

Uncertainties are taken to be the standard deviations of the mean in each spatial direction of each of the detected particle images as described in [25]. This uncertainty estimate is purely statistical and does not distinguish between measurement uncertainty and uncertainty due to tracer movement, nor does it reflect any biases associated with the measurement itself. Measurement biases are currently being explored as part of a separate work. Further discussion of this method, including a comparison of the FPI and G-means methods for PEPT, is found in [11].

### Cell selection

For this study, a cell was needed that can be activated with a positron-emitting isotope. The isotope ^18^F was chosen due to its ready availability and its relatively short half-life of 109.8 minutes. Ideally, a cell would be able to uptake the radioisotope and be unable to export it. To accommodate this, the yeast *Saccharomyces cerevisiae*, strain BY4741-SSY3 (*MATa his3Δ1 leu2Δ0 ura3Δ0 fex1Δ fex2Δ*) [26] which is deleted for the Fluoride EXporter (*FEX*) genes was grown in sterile YEPD broth (1% yeast extract, 2% peptone, 2% glucose) overnight at 30°C with shaking. Deletion of the FEX gene prohibited efflux of ^18^F from the cell. The transporters that modulate influx of fluorine into yeast cells are not known. Cells were harvested by centrifugation, washed twice with water, counted on a hemocytometer, and diluted to a final concentration of 5 x 10^8^ cells/mL in a solution of sterile 2% glucose. Cells were prepared immediately before each uptake experiment and kept chilled until transportation to the activation experiment site. Granulated yeast extract and granulated peptone were obtained from Research Products International Corporation (Mt. Prospect, IL, USA). Glucose was purchased from Fisher Scientific (Fair Lawn, NJ, USA).

### Cell activation and experiment

On the morning of the experiment, the yeast cell suspension (5 x 10^8^ cells/ml prepared as described above) was agitated with a vortex mixer, and 2 μL of this suspension was pipetted into the bottom of a 1.7 mL Eppendorf tube to provide approximately 1 x 10^6^ cells for radiolabeling. A previously prepared solution of 40% glucose was mixed in equal proportions with 0.40 M citrate phosphate buffer, pH 2.5, to provide a buffer solution to maintain a constant pH during the activation. Glucose was added to provide energy for yeast transport mechanisms, and the pH was adjusted to stimulate the uptake of ^18^F [26]. 2 μL of the buffer solution was pipetted into the Eppendorf tube containing the cells, creating an environment of approximately 10% glucose, 0.10 M citrate phosphate buffer at pH 2.5 for cell incubation. The yeast were then brought to the University of Tennessee Medical Center (UTMC) for radiolabeling.

At UTMC 20 μL of aqueous ^18^F ion solution with a specific activity of 63 MBq/μl was introduced into the Eppendorf tube containing the yeast for a total activity of 1.3 GBq, a total volume of 24 μl, and a yeast density of approximately 41,700 cells/μL. The Eppendorf tube was then briefly pulsed in a microfuge to bring down any fluid on the walls of the tube and vortex mixed to ensure good mixture of the yeast suspension and the activation medium. After 8 minutes the activation tube was vortex mixed, and 3 μL of the suspension (125,000±7,000 yeast cells) were pipetted onto the filter of an EMD Millipore Ultrafree-MC centrifugal filter device with a 0.65 μm pore size Durapore membrane for rinsing. The activity of this filter was measured to be 78 MBq. Deionized water was then pipetted onto this yeast-laden membrane and centrifuged at 3000 RCF. The flowthrough was collected, and its activity was measured. This washing procedure was repeated two more times until there was negligible activity in the flowthrough. Upon completion of the washes the remaining activity associated with the filter was 6.88 MBq. Assuming 125,000 yeast cells were on the filter with the remaining activity contained in the yeast cells, and given uncertainties associated with initial cell counting and pipetting, this gives an average activity per cell of 55±3 Bq/cell.

This process of removing cells from the activation medium and washing was repeated two additional times with measured activities being 40±2 Bq/cell for the second sample and 27±2 Bq/cell for the third sample. Because measured activity of the cell samples was decreasing as a function of time, it is expected that the yeast had become saturated with ^18^F. As it had the highest measured activity per cell, the first activated yeast sample was selected for use in the remainder of the experiment.

Yeast cells were then removed from the surface of the filter by placing 200 μL of deionized water into the filter cartridge followed by vortex mixing and transferring of the solution to a clean Eppendorf tube. The filter wash to collect cells was repeated twice, and each wash was pooled into the Eppendorf tube, resulting in a final solution volume of 600 μL. Activity of this suspension was measured found to be 3.7 MBq. At this time, the cell-specific activity of the sample had decayed to 42±3 Bq/cell, indicating the presence of 88,000±5000 cells. This sample of 147±9 cells/μL was then transferred from UTMC to the final test facility at the University of Tennessee. Here, serial dilution was employed to reduce the number density of the sample to 8.6±0.5 cells/μL.

**Fig 2:**
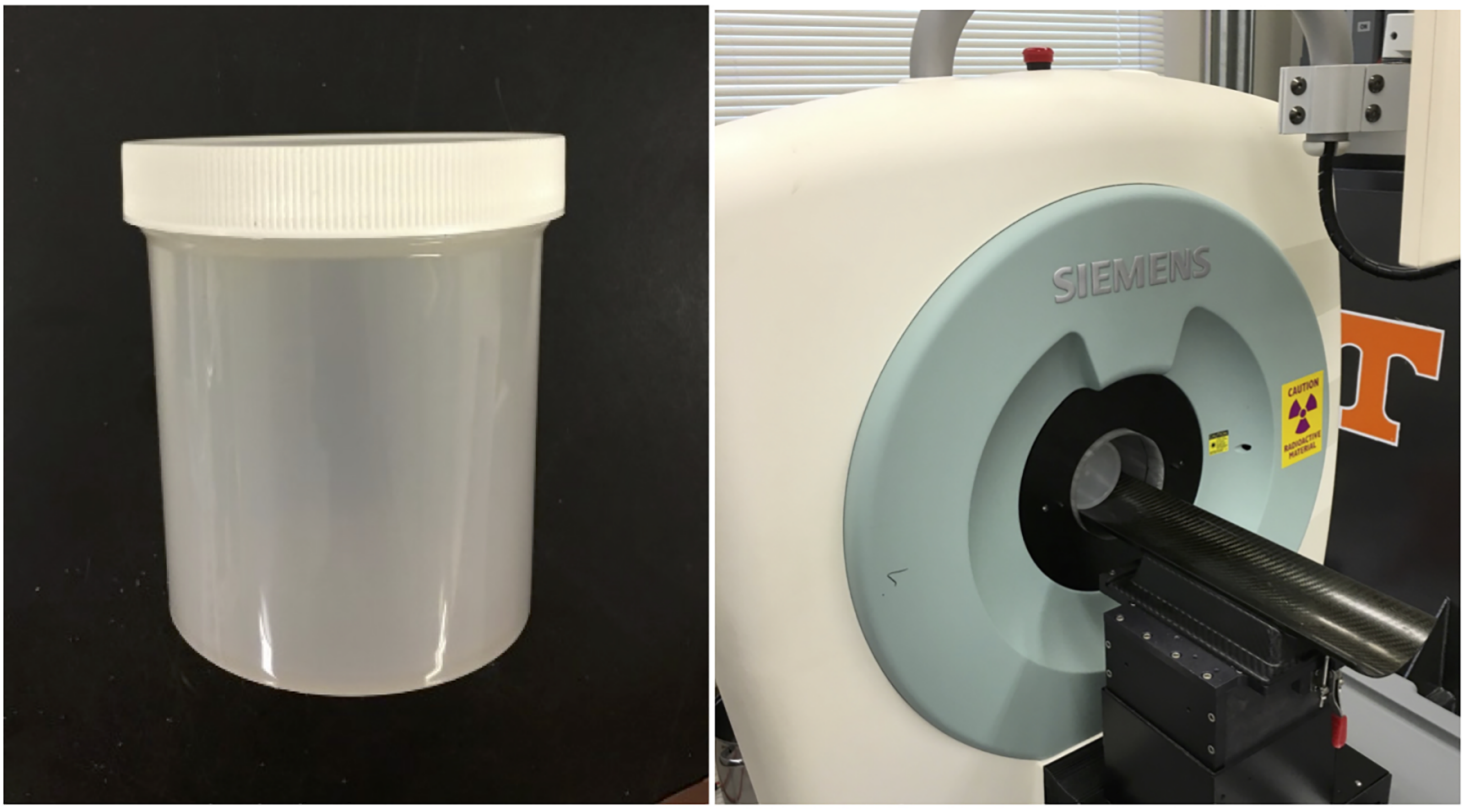
Photographs of experimental equipment. (Left) 500 mL bottle used in cell tracking experiment. (Right) Photograph of bottle placed into bore of PET scanner.

A 2 μL portion of this suspension was then pipetted into a 500 mL cylindrical bottle of deionized water, seen in Fig 2. Given the cell density of the injected suspension and standard counting statistics, it is expected that 17±4 cells were then contained in the bottle. The bottle was then shaken to distribute the cells, and placed in the bore of a Siemens Inveon PET scanner as seen in Fig 2. The bottle was left stationary for 210 s to allow flow to stagnate in the bottle. A PET acquisition scan was begun. At this time, the activity of the cells had decayed to 32±2 Bq/cell. The sample was scanned for 300 s using an energy window of 425–625 keV and a coincidence timing window of 3.438 ns. A second, identical scan was performed immediately after the data archiving of the first scan, with no additional agitation or yeast addition.

The reduction in sensitivity caused by this energy window restriction was tested independently of this experiment using a 0.26 MBq ^22^Na point source. The sensitivity was measured and found to be 5.7% for a 350–650 keV energy window at center of FOV and reduced to 5.2% for a 425–625 keV energy window. Both of these values are lower than the peak sensitivity of 6.72% reported in literature for the Inveon using a 350–625 keV energy, measured using a similar ^22^Na point source [18].

### PEPT processing

In analyzing the experimental data via PEPT, CLs were processed in groups of length 60 s, moved 3 s at each time step (i.e. with 57 s of overlap between time steps). Assuming each of our cells to be of activity 32 Bq, and using the scanner’s peak sensitivity of 5.2%, it is expected that a coincidence event would be detected at a rate of 1.7 events per s per cell. Assuming this to be a Poisson random process, 3 s time steps were chosen because it is at 3 s that the probability of no new event detections falls below 1% for each cell. The 60 s window was chosen because it is at this length that 100 events are expected per cell, a number which experience has shown is sufficient for position triangulation. While it is recognized that use of such overlapping time steps will smooth the individual trajectories, it was deemed beneficial for detecting and linking particle trajectories in this low activity environment as it allowed the use of many CLs at each time step while still maintaining finer time resolution and smaller between frame displacements. CLs were then tallied across a grid of element size 2 mm × 2 mm × 2 mm, and local maxima were calculated using values of *r* = 0.3 and *w* = 4.

## Results

### Cell tracking results

As described in the previous sections, a sample of approximately 125,000 yeast cells was successfully activated to 55±3 Bq/cell. After the completion of the procedure, transfer of the cells, transportation and dilution of the sample, a useable sample of 88,000 yeast cells of activity 32±2 Bq/cell was produced. Of these, 17±4 cells were placed in the FOV of the scanner and tracked.

**Fig 3:**
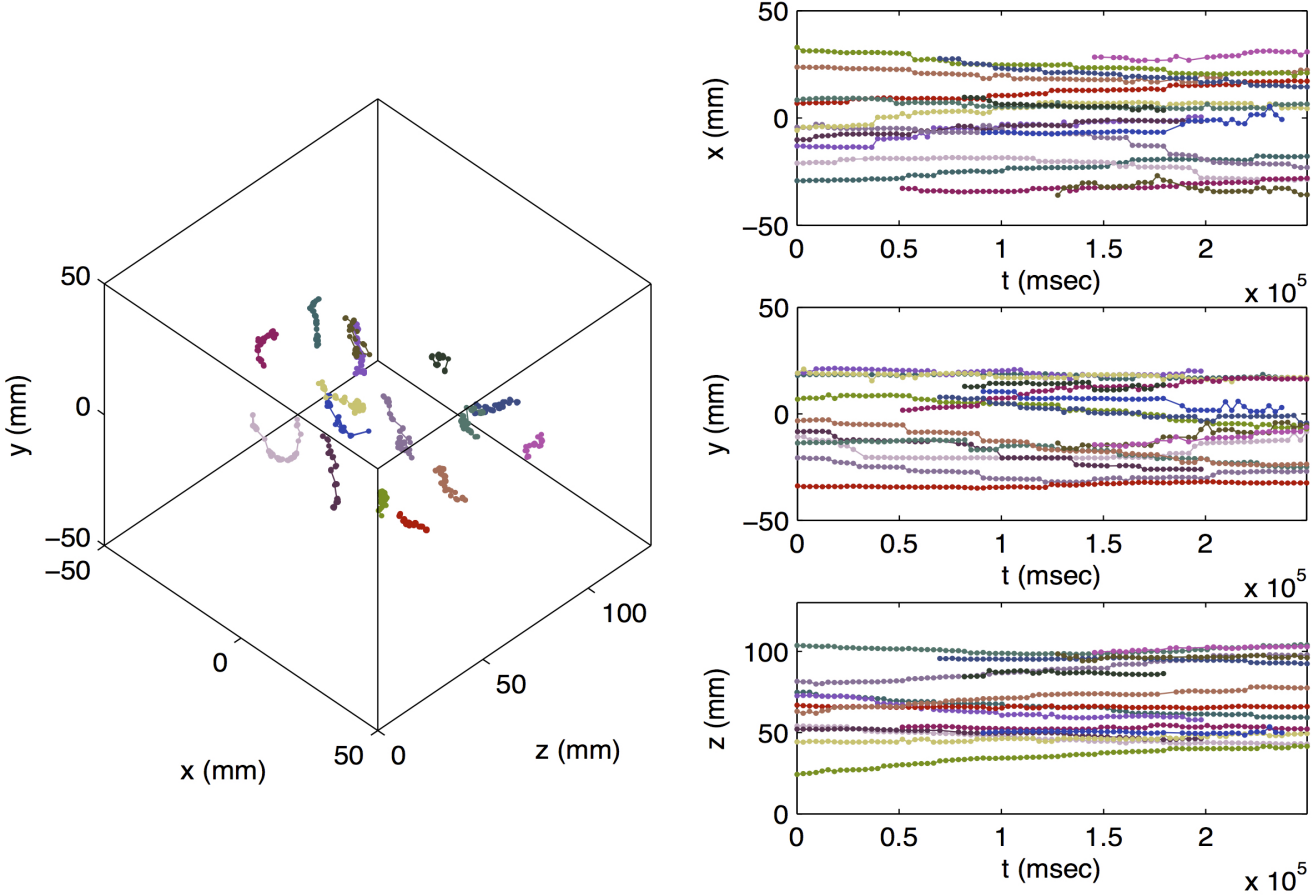
Trajectories of yeast cells measured via PEPT in first scan. Different colors indicate different particles. Colors are coordinated by particle across panels.

**Fig 4:**
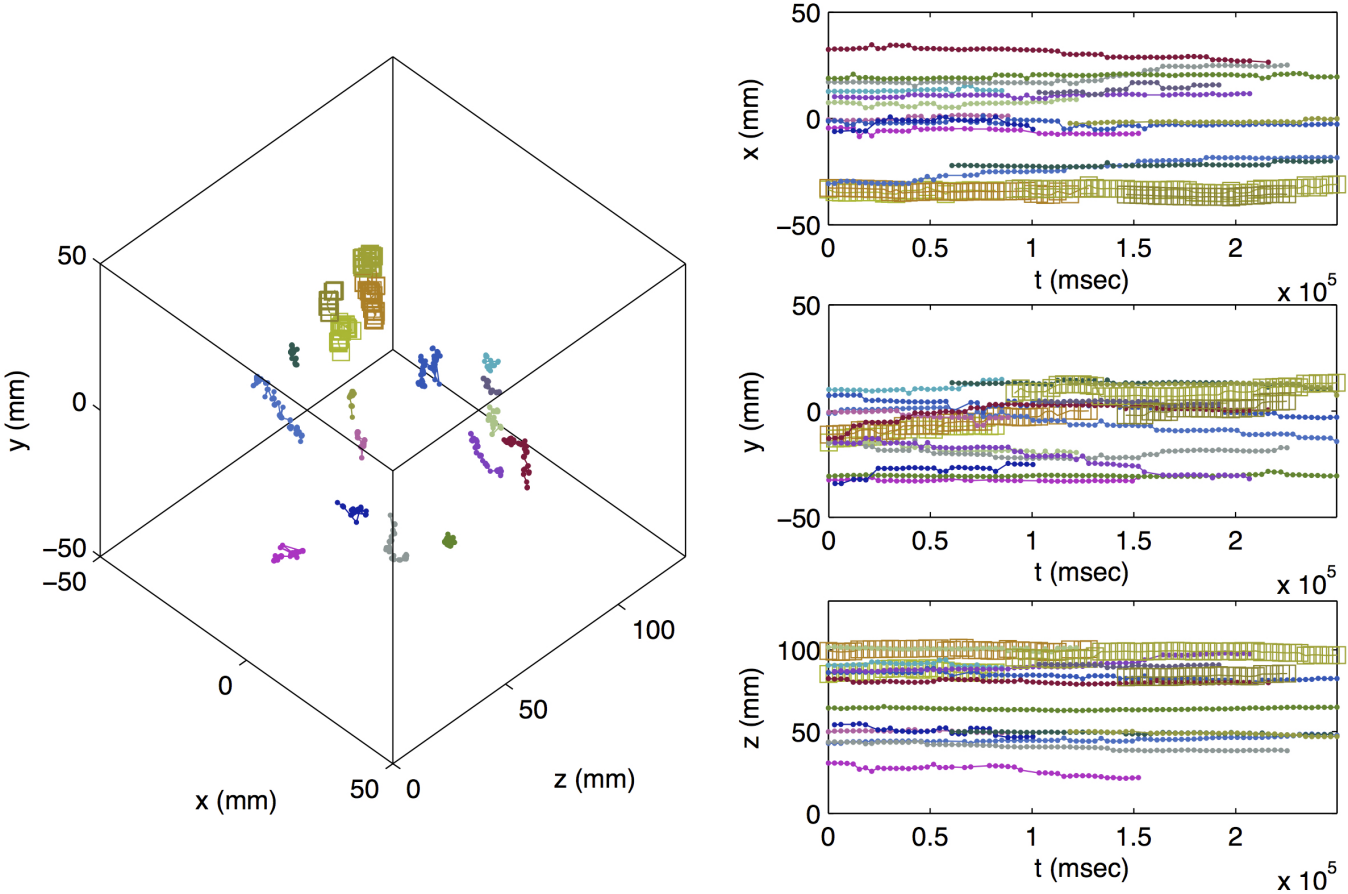
Trajectories of yeast cells measured via PEPT in second scan. Different colors indicate different particles. Colors are coordinated by particle across panels. Trajectories that are suspected to be affected by occlusion are indicated by large square icons.

Fig 3 shows a 3D representation of the cell trajectories measured in the first PEPT experiment, alongside their *x* (radial, horizontal), *y* (radial, vertical), and *z* (axial, horizontal) coordinates as a function of time. Each color represents a different particle, and colors are coordinated by particle across panels. The same is shown for the second scan in Fig 4. In total, 16 cell trajectories were identified during the first scan, and 18 trajectories were identified during the second scan. Average calculated localization uncertainties were 0.56 mm, 0.56 mm, and 0.50 mm in the *x*, *y*, and *z* directions, respectively, in the first scan and 0.58 mm, 0.59 mm, and 0.52 mm in the second scan. Visual inspection of the results of the second scan indicated that there were likely two instances of occlusion (detection, loss, and subsequent detection of the same cell) in the second scan, indicating that only 16 cells were found. Trajectories affected by this are indicated by square markers in Fig 4.

### Count rate assessment

The measured count rate is analyzed to verify that cells were present in the scanner. A background scan was performed for six hours using the 425–625 keV energy window, and a background coincidence count rate of 170 counts per minute was detected. After correcting for this, an average count rate of 1330 events per minute was measured during this experiment. Assuming 16 cells were present in the scanner FOV, as detected, this implies a specific count rate of 83 events per cell per minute, below but similar to the expected event rate of 100±6 per cell per minute. This is expected due to the average sensitivity of the scanner throughout its bore being below the cited peak value. These findings corroborate the presence of roughly 16 activated cells in the scanner volume.

### Individual frame analysis

**Fig 5:**
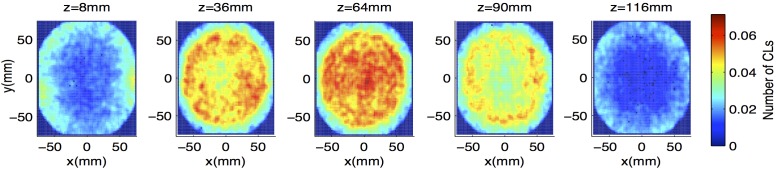
Average background line density grid at 5 axial locations in Inveon scanner. Images show number of CL crossings per minute, with line crossings counted on a 2 mm × 2 mm × 2 mm grid. Images are smoothed via a boxcar kernel of side width 3 voxels before averaging.

It is recognized that this detection and tracking are performed using activity well below that often used in conventional PET [20]. For this reason, an individual image frame from this experiment is considered. The intrinsic background random coincident count rate from ^176^Lu decay results in CLs throughout the image volume, with a maximum random CLs density at the center of the image volume. However, these tend to be distributed throughout the FOV and rarely form clusters that could be mistaken for particles. Fig 5 shows five axial slices of a 2 mm × 2 mm × 2 mm line density grid taken over a six hour background scan. The grid has been normalized to show number of CL crossings per minute and smoothed with the same boxcar kernel used in this experiment. Here one can see that average background in the scanner is distributed throughout its volume and stays below a fraction of a CL per voxel throughout.

**Fig 6:**
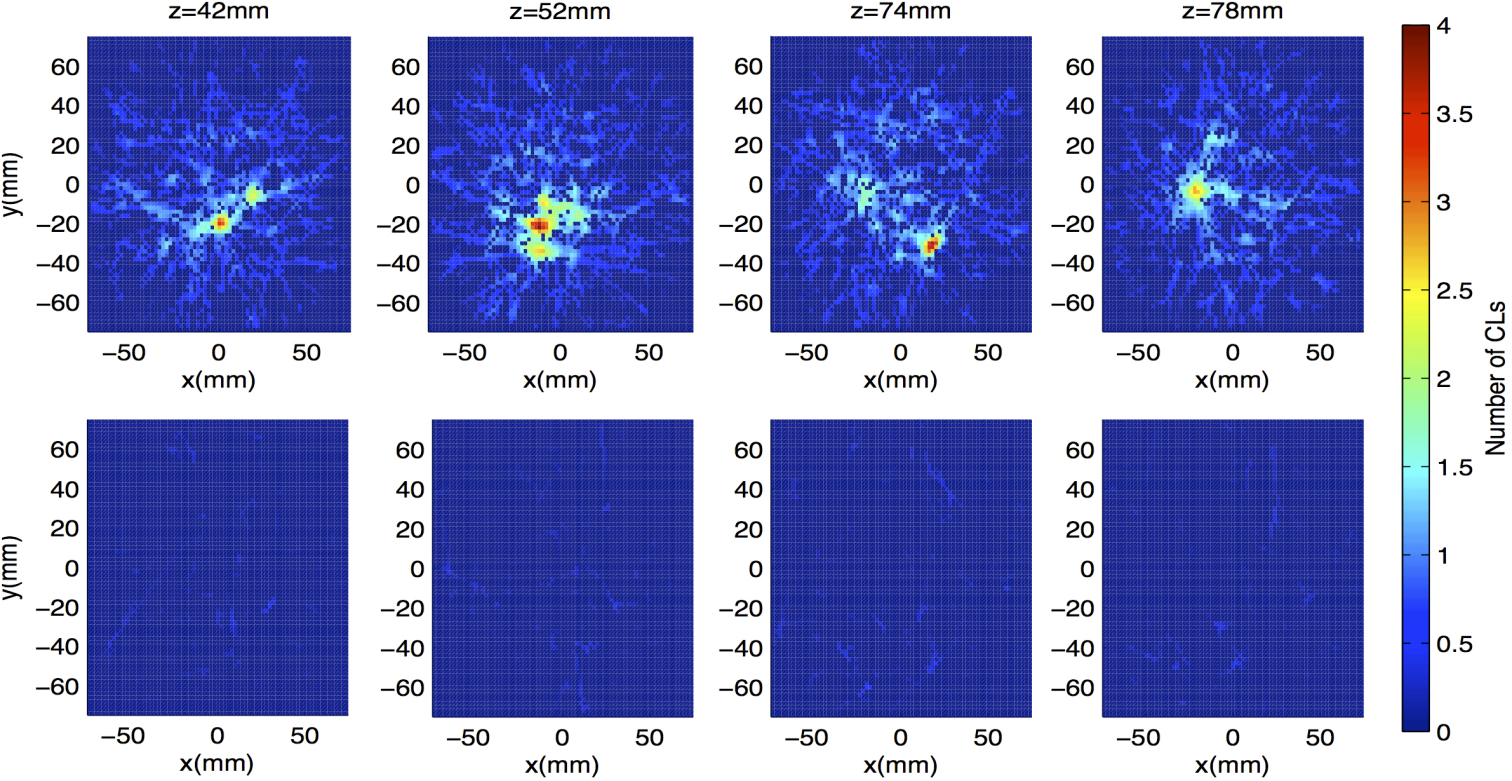
Sample line density grid images. (Top Row) Images from first minute of yeast tracking experiment. Four axial locations show particle images. CL crossings are counted on a 2 mm × 2 mm × 2 mm grid, and images are smoothed via a boxcar kernel of side width 3 voxels. (Bottom Row) For comparison, images at same axial positions from first minute of background scan are shown.

In contrast to Fig 5, the top row of Fig 6 shows four axial slices from the smoothed line density grid taken in the first minute (first time step) of the first yeast scan. Axial slices are chosen such that at least one cell image is seen in each. Here, the regions of concentrated line density corresponding to cells are easily distinguished from random background fluctuations.

Data from the background scan were also considered in 60 s time steps. In each time step, CL crossings were counted across a 2 mm × 2 mm × 2 mm grid, and this grid was smoothed as done in this experiment. Sample image slices from the first minute of the background scan are seen in the bottom row of Fig 6. In this set, the average peak value of line density across frames was 0.56 lines per voxel. For the experimental data, the average peak value of smoothed line density was 4.5 CLs per voxel. As such, a signal-to-noise ratio of about 8 is observed on average in this measurement.

### Cell viability

Fluoride’s toxicity to yeast cells is known [26]. In prior work with the strain of yeast used herein, Sanshu et al. saw an inhibition of cell growth in micromolar concentrations of fluoride. However, there was little observed difference between cell growth rates in 0 μM and 20 μM fluoride solutions, with no noticeable effects for exposures less than six hours [26]. Based on the initial 63 MBq/μl activity of our ^18^F solution, the cells used in this experiment were exposed to a 1 μM fluoride solution for less than 10 minutes, indicating that cell death due to fluoride poisoning was unlikely.

Cell death due to radiation exposure is also a concern in this work. Monte Carlo simulations were carried out in MCNP6 to estimate the dose received by the cells in our experiment. In these simulations, the cell was treated as a sphere of water of 2.15 μm radius centered in a cylinder of water with a height of 5.89 cm and a radius of 1.06 cm, roughly approximating the geometry and volume of fluid used during the yeast activation experiment. In the first simulation, an ^18^F source was distributed within the simulated yeast cell to estimate dose from radiation originating within the activated cell. In the second simulation, a source of ^18^F was distributed throughout the water cylinder to simulate dose from the activation procedure. In each case, energy deposition in the simulated cell was tallied to estimate the dose to the cell on a per decay basis. This was then used to estimate the dose to the activated cells as a function of time. Based on the MCNP results, the 8 minute activation period, and the 86 minute interval of time between cell activation and the initiation of scanning, it is estimated that the cells received doses of 833±29 Gy from extracellular radiation sources and 402±21 Gy from intracellular sources. Cited uncertainties reflect statistical uncertainty of MCNP6 and uncertainty in activities. These doses correspond to 4.0×10^−10^ and 0.16 percent of the total energy deposited in the system by each type of source, respectively, indicating that the vast majority of energy deposition occurred outside the cells. Game et al. [27] observed the survival rate of yeast cells exposed to x-ray irradiation to fall below 1% at about 1200 Gy. Considering the small population of cells tracked in this experiments (16) and the estimated total dose of roughly 1200 Gy, it is unlikely that a significant number of cells, if any, maintained the ability to reproduce; however, it is possible that cells remained alive and functioning during the test period. It is suggested that future experiments be performed with less activity or shorter activation periods to reduce the dose received by the cells to increase the likelihood of tracking living cells. Furthermore, a biological assay could be performed to check the viability of the yeast post-irradiation. No direct viability study was performed in this test as the focus of this study was tracking of the radiolabelled cells.

## Discussion

This work shows that it is possible to track multiple cells at once utilizing PEPT. Previous work has shown that it is possible to track a single cell utilizing PEPT [17, 19]; however, this left the challenge associated with isolating a single radiolabeled cell from a host of activated cells. This work addresses that issue by removing the requirement that only a single cell may be within the field of view of the scanner at any one time. Nevertheless, there are limitations for this method.

It is of note that for the PEPT processing settings used herein, no two cells could be resolved within a separation of roughly 1 cm. Care must be taken in sample preparation to ensure that cell number density is such that cells are amply separated. In the case that two or more cells are located within this resolving limit, the most active cell will be tracked with a biasing of its measured position toward the center of this multiple cell system. If the nearby cells are of very similar activity, it is possible for the cells to be confused and switched during the tracking process. A brief discussion of this effect is found in [11]. If a sample cannot be prepared with well-separated cells, an application must be chosen such that occasional confusion of tracked cells will not detrimentally affect results.

Furthermore, in this work, the exact number and location of cells present in the sample are unknown. The number of cells detected by PEPT is within the uncertainty of the number of cells in the sample expected from serial dilution. This number is further corroborated by the count rate assessment described herein. To further assess performance of this method, it is suggested that simulations be performed so that measured particle positions can be compared to known input locations. A GATE [28] model of the Siemens Inveon exists [29], but this model has been seen to overpredict the number of events detected for a given activity when compared to experiment. A similar issue has been observed in other GATE Inveon models when applied to point source tracking [17, 30]. Work is ongoing to understand the reasons for these discrepancies and enable reliable GATE modeling for point source tracking. Nonetheless, the observation of trajectories stretching across many frames indicates that measured trajectories are not likely due to noise. Past work has shown that no false positive trajectories were observed when accepted trajectories were limited to those at least 20 time steps in length [11]. In this work, the shortest observed trajectory is 27 frames in length. It is possible that other cells were present but not able to be detected.

While this work shows the capability of the activating and tracking of yeast cells, future work should be performed to extend this technique to cells of medical or biological interest. This work should also include a study on the viability of the cells via direct inspection to understand any inhibition of their functions due to exposure to radiation. Mammalian cells can take up fluorine ions, but the transport mechanism(s) is not known. There are known homologs of FEX in animal cells, although their presence in mammalian cells has not yet been determined [26]. Therefore, labeling efficiency would need to be determined for specific cell types as accumulation in various cell types would differ significantly. Past studies have shown that mammalian cells can be radiolabeled with ^18^F in the form of fluorine compounds to activities that can be tracked using this method and remain alive. Ma *et al*. [16] used hexadecyl-4-[^18^F] fluorobenzoate ([^18^F]HFB) to activate a population of 5×10^5^ rat mesenchymal stem cells to 18 Bq/cell. Zhang *et al*. [15] used the same radioligand to activate a population of 2×10^6^ human circulating progenitor cells to 22–30 Bq/cell. In both studies, cells were seen to be 90% viable after 4 hours with at least 88% activity retention. Based on the results herein, cells of this activity could be tracked individually if a small population (approximately 20) was isolated. *In vivo* tracking of such cells could provide novel insight into individual cell dynamics in stem cell therapies and allow for monitoring of the administration of such therapies [15]. If suitable radiolabeling techniques can be engineered, this method could be extended to the tracking of leukocytes for study of infection and inflammation [31], cancer cells for study of migration and metastasis [32], and a number of other cells of interest for cell-based therapy [33].

## Conclusion

A method for multiple particle tracking via PEPT is presented based on optical feature point identification methods. This method was tested wherein a population of 125,000 yeast cells was activated by ^18^F uptake, and 16 of these cells with activity 32 Bq/cell were tracked using multi-PEPT to near 1 mm accuracy. To the authors’ knowledge this is the first experimental realization of individual cell tracking using positron imaging techniques. Further work is underway to determine the limits and uncertainties associated with this tracking method using both experiment and simulation via GATE. It is desired that the existing GATE model be corrected so that simulation can be used to direct future cell tracking endeavors involving PEPT.

## Supporting information

S1 File Crystal data for background scan. Coincidence event locations for 6 hour background scan. Each row is a separate coincidence event. Column 1 is time mark (in ms). Columns 2–4 are the *x*, *y*, and *z* locations (in mm), respectively, for one detector crystal in the coincidence event. Columns 5–7 are the *x*, *y*, and *z* locations, respectively, for the other detector crystal in the coincidence event.
(TXT)

S2 File Crystal data for Run 1. Coincidence event locations for Run 1 of cell tracking experiment. Each row is a separate coincidence event. Column 1 is time mark (in ms). Columns 2–4 are the *x*, *y*, and *z* locations (in mm), respectively, for the one detector crystal in the coincidence event. Columns 5–7 are the *x*, *y*, and *z* locations, respectively, for other detector crystal in the coincidence event.
(TXT)

S3 File Crystal data for Run 2. Coincidence event locations for Run 2 of cell tracking experiment. Each row is a separate coincidence event. Column 1 is time mark (in ms). Columns 2–4 are the *x*, *y*, and *z* locations (in mm), respectively, for the one detector crystal in the coincidence event. Columns 5–7 are the *x*, *y*, and *z* locations, respectively, for other detector crystal in the coincidence event.
(TXT)

S4 File Trajectories from Run 1. Compressed folder contains text files describing individual measured trajectories from Run 1 of cell tracking experiment. Each file corresponds to a different measured trajectory. Columns 1–4 indicate the time (ms) and *x*, *y*, and *z* positions (mm) of each detection. Columns 5–7 are calculated uncertainties in *x*, *y*, and *z* directions (mm), respectively.
(ZIP)

S5 File Trajectories from Run 2. Compressed folder contains text files describing individual measured trajectories from Run 2 of cell tracking experiment. Each file corresponds to a different measured trajectory. Columns 1–4 indicate the time (ms) and *x*, *y*, and *z* positions (mm) of each detection. Columns 5–7 are calculated uncertainties in *x*, *y*, and *z* directions (mm), respectively.
(ZIP)
